# Stroke After Transcatheter Aortic Valve Replacement With Contemporary Valves: Insights From the OCEAN-TAVI Registry

**DOI:** 10.1016/j.shj.2026.101099

**Published:** 2026-07-22

**Authors:** Yusuke Watanabe, Masanori Yamamoto, Hirofumi Hioki, Masafumi Saito, Jo Omiya, Kento Kito, Yasuyuki Tsuchida, Yosei Iseki, Masataka Arakawa, Mizuki Miura, Akihisa Kataoka, Takahiro Tokuda, Shinichi Shirai, Kenichi Ishizu, Toru Naganuma, Futoshi Yamanaka, Masahiko Noguchi, Yohei Ohno, Tetsuro Shimura, Fumiaki Yashima, Gaku Nakazawa, Masahiko Asami, Masaki Izumo, Hidetaka Nishina, Hiroshi Ueno, Hiroto Suzuyama, Kazumasa Yamasaki, Kenji Nishioka, Daisuke Hachinohe, Yasushi Fuku, Toshiaki Otsuka, Kentaro Hayashida, OCEAN-TAVI investigators

**Affiliations:** a Department of Cardiology, Teikyo University School of Medicine, Tokyo, Japan; b Department of Cardiology, Toyohashi Heart Center, Toyohashi, Japan; c Department of Cardiology, Nagoya Heart Center, Nagoya, Japan; d Department of Cardiology, Gifu Heart Center, Gifu, Japan; e Department of Cardiology, IMS Tokyo Katsushika General Hospital, Tokyo, Japan; f Department of Cardiology, Kokura Memorial Hospital, Kokura, Japan; g Department of Cardiology, New Tokyo Hospital, Matsudo, Japan; h Department of Cardiology, Shonan Kamakura General Hospital, Kamakura, Japan; i Department of Cardiology, Tokyo Bay Urayasu Ichikawa Medical Center, Urayasu, Japan; j Department of Cardiology, Tokai University School of Medicine, Isehara, Japan; k Department of Cardiology, Saiseikai Utsunomiya Hospital, Tochigi, Japan; l Department of Cardiology, Kindai University, Osaka, Japan; m Division of Cardiology, Mitsui Memorial Hospital, Tokyo, Japan; n Department of Cardiology, St. Marianna University School of Medicine, Kawasaki, Japan; o Department of Cardiology, Tsukuba Medical Center Hospital, Tsukuba, Japan; p Department of Cardiology, Toyama University Hospital, Toyama, Japan; q Department of Cardiology, Saiseikai Kumamoto Hospital, Kumamoto, Japan; r Department of Cardiology, Sapporo Higashi Tokushukai Hospital, Sapporo, Japan; s Department of Cardiology, Hiroshima City Hiroshima Citizens Hospital, Hiroshima, Japan; t Department of Cardiology, Sapporo Heart Center, Sapporo Cardiovascular Clinic, Sapporo, Japan; u Department of Cardiology, Kurashiki Central Hospital, Okayama, Japan; v Department of Hygiene and Public Health, Nippon Medical School, Tokyo, Japan; w Center for Clinical Research, Nippon Medical School Hospital, Tokyo, Japan; x Department of Cardiology, Keio University School of Medicine, Tokyo, Japan; y Department of Medicine II, Kansai Medical University, Osaka, Japan

**Keywords:** Cerebral embolic protection, Stroke, Transcatheter aortic valve implantation (TAVR)

## Abstract

**Background:**

Stroke remains a serious complication after transcatheter aortic valve replacement (TAVR), even with contemporary devices. The objective of the study was to evaluate the incidence, predictors, and prognosis of in-hospital stroke in patients undergoing TAVR with contemporary valve platforms.

**Methods:**

We analyzed 5123 patients from the multicenter OCEAN-TAVI registry who underwent TAVR with SAPIEN 3 Ultra RESILIA, Evolut FX, or Navitor between May 2022 and December 2024. Stroke incidence across valve types was compared using inverse probability of treatment weighting (IPTW). Predictors were assessed using multivariable Firth penalized logistic regression.

**Results:**

In-hospital stroke occurred in 2.5% of patients. After IPTW adjustment, stroke incidence differed among valve types: 1.7% of patients treated with SAPIEN 3 Ultra RESILIA (S3UR), 4.5% with Evolut FX, and 2.4% with Navitor (*p* = 0.044). Disabling stroke was more frequent with Evolut FX (2.5%) compared with S3UR (0.4%) and Navitor (0.7%; *p* < 0.001). Self-expanding valve use (adjusted odds ratio [aOR] 2.64, 95% CI 1.67–4.16 for Evolut FX; aOR 2.37, 95% CI 1.35–4.18 for Navitor; both vs. S3UR), prior stroke (aOR 1.83, 95% CI 1.19–2.73), reduced renal function, and nontransfemoral access (aOR 2.11, 95% CI 1.29–3.45) were independently associated with in-hospital stroke. One-year survival was significantly lower among patients with stroke than among those without stroke (73.0% vs. 90.3%; *p* < 0.001).

**Conclusions:**

In-hospital stroke remains an important complication after contemporary TAVR and is associated with worse clinical outcomes. Differences in stroke incidence across valve types were observed, highlighting the need for improved risk assessment.

## Highlights

• In-hospital stroke occurred in 2.5% of patients after contemporary transcatheter aortic valve replacement.
• Evolut FX was associated with higher stroke risk than Sapien 3 Ultra RESILIA after inverse probability of treatment weighting adjustment.
• Disabling stroke rates differed significantly across valve types after adjustment.
• A four-variable risk score identifies patients at elevated stroke risk after transcatheter aortic valve replacement.
• In-hospital stroke was associated with significantly worse 1-year survival.

## Graphical Abstract

**Figure:**
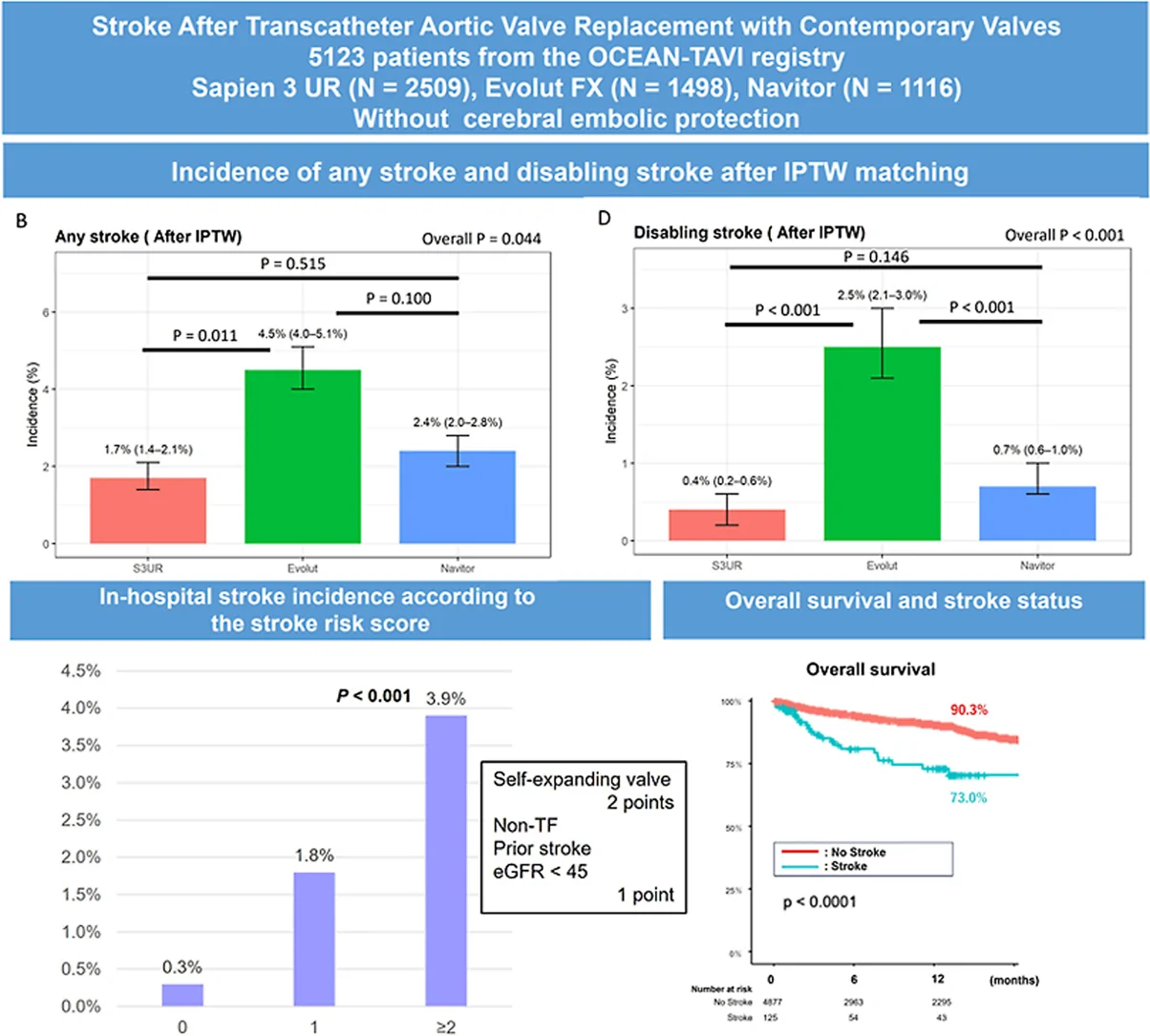

## Introduction

Transcatheter aortic valve (TAV) replacement (TAVR) has become the standard treatment for patients with severe aortic stenosis (AS) across all surgical risk categories.^1^^,^^2^ With improvements in valve design and procedural technique, periprocedural complications have steadily declined.^3^, ^4^, ^5^, ^6^ Nevertheless, ischemic stroke remains one of the most devastating adverse events following TAVR; it is associated with increased mortality, prolonged hospitalization, and impaired long-term functional outcomes.

In response to this persistent risk, cerebral embolic protection (CEP) devices have been developed to reduce embolic load during valve deployment. However, large, randomized trials such as PROTECTED TAVR and BHF PROTECT-TAVI^7^^,^^8^ have shown no significant reduction in overall stroke rates with routine CEP use. These findings have shifted the clinical conversation toward identifying high-risk subgroups who may derive selective benefit from CEP.

In this study, using data from the Optimized CathEter vAlvular iNtervention–Transcatheter Aortic Valve Implantation (OCEAN-TAVI) multicenter Japanese registry, we aimed to evaluate the incidence, predictors, and clinical consequences of in-hospital stroke following TAVR with 3 widely used contemporary devices: SAPIEN 3 Ultra RESILIA (S3UR) (Edwards Lifesciences, Irvine, California), Evolut FX (Medtronic, Minneapolis, Minnesota), and Navitor (Abbott, Santa Clara, California).

## Methods

### Study Population

The data used in this study were extracted from the OCEAN-TAVI registry database. This study was registered with the University Hospital Medical Information Network Clinical Trials Registry (UMIN000020423) and approved by the International Committee of Medical Journal Editors. The study protocol was designed in accordance with the Declaration of Helsinki and approved by the ethics committees of all participating hospitals. Informed consent was obtained from all patients before their enrollment in the study.

The study was conducted with a cohort of 6121 patients who were enrolled in the OCEAN-TAVI registry between May 2022 and December 2024 at the participating 22 Japanese institutes. Of the 6121 patients initially identified from the OCEAN-TAVI registry, 998 were excluded because of hemodialysis dependence (n = 802) and/or a history of TAV-in-surgical aortic valve or TAV-in-TAV (n = 227), leaving a final study population of 5123 TAVR patients for analysis. Of these, 2509 (49.0%) received a S3UR, 1498 (29.2%) received an Evolut FX, and 1116 (21.8%) received a Navitor. A flowchart of the study is shown in Figure 1.

**Figure 1:**
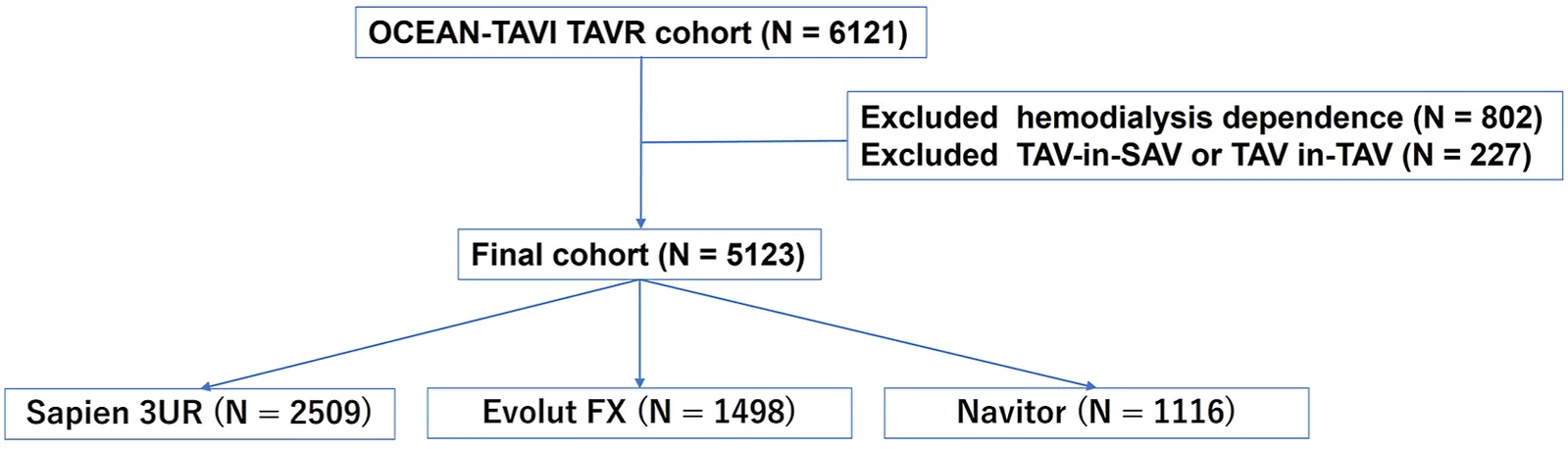
**Study flowchart.** Among 6121 patients who underwent TAVR in the OCEAN-TAVI registry, 998 were excluded due to hemodialysis dependence or a history of TAV-in-SAV or TAV-in-TAV. The final study population consisted of 5123 patients treated with S3UR, Evolut FX, or Navitor. Abbreviations: OCEAN-TAVI, Optimized CathEter vAlvular iNtervention–Transcatheter Aortic Valve Implantation; S3UR, SAPIEN 3 Ultra RESILIA; SAV, surgical aortic valve; TAV, transcatheter aortic valve; TAVR, transcatheter aortic valve replacement.

All enrolled patients underwent TAVR using the latest valves: S3UR, Evolut FX, or Navitor. Prosthesis type and size were determined by a local heart team based on preprocedural echocardiography and multidetector computed tomography (CT) findings. No CEP devices were used during the procedures. All patients underwent standard 2-dimensional and Doppler transthoracic echocardiography before and after the procedure, and conventional parameters were measured according to the guidelines of the American Society of Echocardiography^9^ by experienced echocardiographers in each hospital.

### Data Definition and Study Endpoints

The OCEAN-TAVI database included baseline patient characteristics, laboratory data, transthoracic echocardiography findings, and CT parameters. The severity of AS was determined by the aortic valve area, indexed aortic valve area, or mean pressure gradient. All procedural complications, including stroke, were evaluated and defined using Valve Academic Research Consortium-3 (VARC-3) criteria.^10^ The primary endpoint was any in-hospital stroke, defined as any new neurologic deficit of vascular origin occurring during the index hospitalization. Disabling stroke was defined as a stroke resulting in significant persistent disability (typically a modified Rankin Scale ≥2 at 90 days). Secondary neurologic endpoints included stroke subtypes (i.e., ischemic vs. hemorrhagic stroke) and transient ischemic attack (TIA) occurring before hospital discharge. All events were identified through chart review and adjudicated by the treating clinical teams per standard criteria.

### Statistical Analysis

Baseline characteristics were summarized as mean ± SD for continuous variables and counts (percentages) for categorical variables. Groupwise comparisons among the 3 valve cohorts were performed using analysis of variance or Kruskal–Wallis tests for continuous variables and chi-square tests for categorical variables, as appropriate. To account for potential selection bias in device type, we used inverse probability of treatment weighting (IPTW) to create a weighted cohort with improved covariate balance. Propensity scores for each valve type were estimated using a multinomial logistic regression model incorporating a comprehensive set of preprocedural and procedural covariates. Stabilized IPTW weights were then calculated and applied to each patient to create a pseudopopulation in which baseline characteristics were similar across the S3UR, Evolut FX, and Navitor groups. Missing baseline data, if present, were addressed with multiple imputation by chained equations (MICE) before weight estimation. Missing data were present in a minority of variables. The variables with the highest proportions of missing data were systolic pulmonary artery pressure (25.4%), left ventricular ejection fraction by modified Simpson method (14.3%), and Society of Thoracic Surgeons (STS) score (4.2%). All other variables included in the propensity score model had <2% missing data. Missing values were addressed using MICE with m = 20 imputations. To account for residual imbalance in procedural covariates after IPTW, doubly robust estimation was additionally performed by applying multivariable logistic regression to the IPTW-weighted cohort with missing data handled by MICE (m = 20). Results were pooled using Rubin's rules.

Covariate balance after weighting was assessed by calculating standardized mean differences (SMDs) for each variable; an absolute SMD <0.1 was considered indicative of acceptable balance between groups. A Love plot illustrating covariate balance before and after IPTW is presented in Supplemental Figure 1. After IPTW adjustment, we compared in-hospital stroke rates across valve types using weighted contingency table analyses and weighted logistic regression for hypothesis testing (Supplement 1). In addition to the IPTW-based comparative analyses, we performed a separate prediction analysis to identify clinical and procedural factors associated with in-hospital stroke. Given the relatively low incidence of stroke events, Firth penalized logistic regression was applied to reduce small-sample bias. Univariable Firth logistic regression analyses were first conducted for descriptive purposes. Subsequently, a multivariable Firth logistic regression model was constructed without IPTW weighting to reflect real-world clinical decision-making. Covariates were selected a priori based on clinical relevance and previous literature rather than univariable screening. Adjusted odds ratios (ORs) with 95% CIs were reported. To develop a clinically applicable stroke risk score, candidate predictors were selected based on prior evidence and results from previous multivariable analyses. The following variables were included in the core model: self-expanding valve (SEV) use, nontransfemoral access, estimated glomerular filtration rate (eGFR) <45 mL/min/1.73 m^2^, and history of prior stroke. Intraprocedural antithrombotic therapies were considered treatment decisions made during the procedure rather than baseline patient characteristics and were therefore not included in the primary multivariable model to avoid overadjustment and confounding by indication. Regression coefficients from the multivariable model were used to derive a points-based scoring system. Points were assigned proportionally to the magnitude of each coefficient and subsequently simplified to enhance clinical usability. Model discrimination was assessed using the area under the receiver operating characteristic curve. Internal validation was performed using bootstrap resampling with 1000 iterations. Calibration was evaluated by comparing observed and predicted event rates across deciles of predicted risk. For clinical interpretation, patients were stratified into 3 risk categories based on the simplified score: 0 points, 1 point, and ≥2 points. The incidence of in-hospital stroke across risk categories was compared using the chi-square test, and Fisher exact test was additionally performed when appropriate. Event rates with 95% CIs were estimated using Wilson method. A 2-sided *p* value < 0.05 was considered statistically significant. Kaplan–Meier curves were constructed to compare survival between patients with and without in-hospital stroke and among patients with in-hospital stroke stratified by valve type. Differences in survival distributions were assessed using the log-rank test for unadjusted analyses. One-year survival probabilities and corresponding 95% CI were estimated from the Kaplan–Meier curves. All tests were 2-tailed, with a *p*-value <0.05 defining statistical significance. Statistical analyses were performed using IBM SPSS software version 29 (IBM Corp., Armonk, New York) and R statistical software (version 4.4.2; R Foundation for Statistical Computing, Vienna, Austria).

## Results

### Baseline Characteristics

Baseline clinical and procedural characteristics of the 3 valve cohorts (before IPTW adjustment) are summarized in Table 1. Significant differences were observed among the S3UR, Evolut FX, and Navitor groups before weighting. Patients treated with the Navitor were slightly older on average than those receiving S3UR (85.8 ± 5.2 vs. 84.7 ± 5.8 years, *p* < 0.001) and were more often females (only 28.9% male in Navitor vs. 36.9% male in S3UR 3, *p* < 0.001). Navitor recipients had smaller body size metrics on average (mean body surface area 1.43 ± 0.17 m^2^ vs. 1.48 ± 0.18 m^2^ in S3UR, *p* < 0.001; body mass index ∼22.0 vs. 22.5 kg/m^2^, *p* = 0.001). The prevalence of major comorbidities varied across groups, although absolute differences were modest. For example, diabetes mellitus was less common in the Evolut FX cohort (25.8%) compared to S3UR (30.9%) and Navitor (28.5%; *p* < 0.001). Chronic obstructive pulmonary disease was also least frequent in Evolut FX patients (5.9% vs. 8.3% in S3UR, *p* = 0.020). Prior cardiac interventions showed some imbalance: the Navitor group had a higher rate of prior pacemaker implantation (7.5%) than S3UR (4.2%) or Evolut (4.5%) (*p* < 0.001), whereas prior coronary artery bypass grafting surgery was slightly more common in Navitor and S3UR patients (∼24%–27%) than in Evolut (21.2%) (*p* = 0.003). The incidences of atrial fibrillation (AF) differed as well (19.1% Navitor vs. 23.9% S3UR, *p* = 0.006), whereas STS predicted risk scores were similar between groups (mean ∼8% in each, *p* = 0.182). Renal function was comparable across cohorts (mean eGFR ∼51 mL/min/1.73 m^2^, *p* = 0.775), and there was no significant difference in prior stroke history (∼10% in all groups, *p* = 0.112). There were also notable anatomic and procedural differences before adjustment (Table 1). Navitor recipients tended to have smaller aortic annuli: the mean annular area was 390.4 ± 65.8 mm^2^ in the Navitor group versus 416.1 ± 78.7 mm^2^ in S3UR (*p* < 0.001; SMD 0.232), consistent with generally smaller annular perimeter (71.4 ± 5.9 mm vs. 73.8 ± 6.9 mm, respectively; *p* < 0.001). Baseline echocardiographic severity of AS differed slightly, patients receiving Evolut FX had a higher mean aortic gradient (49.2 ± 17.4 mmHg) compared to S3UR patients (43.5 ± 15.7 mmHg) and Navitor patients (46.0 ± 15.6 mmHg, *p* < 0.001), despite similar valve areas. Bicuspid aortic valve morphology was more frequently present in the Evolut group (5.8%) than in S3UR (3.2%) and was especially rare in Navitor patients (0.9%, *p* < 0.001). In terms of procedural profile, virtually all cases were performed via the transfemoral approach in each group (>94%), although the Navitor group had the highest rate (97.4% vs. 94.7% in S3UR; *p* < 0.001). Balloon aortic valvuloplasty usage differed markedly by valve type: predilation was performed in only 15.7% of S3UR cases, compared with 79.7% of Evolut FX cases and 95.4% of Navitor cases (*p* < 0.001). Conversely, postdilation was more frequently required in S3UR procedures (16.7%) than in Evolut FX (11.7%) or Navitor (9.0%) (*p* < 0.001). Intraprocedural antithrombotic therapy is summarized in Table 1. Any antithrombotic agent was used in 56.0%, 57.4%, and 63.6% of patients in the S3UR, Evolut FX, and Navitor groups, respectively (*p* < 0.001). Among antiplatelet agents, aspirin and clopidogrel were the most commonly used across all groups, with Navitor recipients receiving clopidogrel more frequently (21.4% vs. 12.4% in S3UR; *p* < 0.001). Among anticoagulants, edoxaban was the most prevalent agent (12.1%, 11.7%, and 9.1%, respectively; *p* = 0.031). The overall distribution of antithrombotic regimens was broadly similar across valve types. New-onset AF during hospitalization occurred in 138 patients (2.7% overall), with rates of 2.4%, 3.2%, and 2.6% in the S3UR, Evolut FX, and Navitor groups, respectively (*p* = 0.335). These preweighting imbalances underscore the differing patient profiles and procedural practices associated with each valve technology. After applying IPTW, baseline differences between the groups were greatly attenuated (Table 2). The weighted effective sample sizes were 5182 for S3UR, 5418 for Evolut FX, and 5935 for Navitor, reflecting the stabilized weights. After IPTW adjustment, baseline differences between groups were substantially reduced. Although most covariates achieved acceptable balance (absolute SMD <0.1), some variables remained modestly imbalanced (SMD >0.1) (Table 2).

**Table 1:** Baseline clinical characteristics of the study population

	Before stabilized IPTW			*p-*value	Test SMD
	S3UR (n = 2509)	Evolut FX (n = 1498)	Navitor (n = 1116)		
Age, y	84.7 ± 5.8	85.0 ± 5.3	85.8 ± 5.2	<0.001	0.129
Sex, male	927 (36.9)	490 (32.7)	322 (28.9)	<0.001	0.115
BSA, m^2^	1.48 ± 0.18	1.46 ± 0.18	1.43 ± 0.17	<0.001	0.195
BMI, kg/m^2^	22.5 ± 3.9	22.3 ± 3.7	22.0 ± 3.6	0.001	0.091
Clinical frailty score	3.7 ± 1.2	3.8 ± 1.2	3.8 ± 1.2	0.013	0.061
Hypertension	1993 (79.4)	1148 (76.6)	910 (81.5)	0.008	0.081
Diabetes mellitus	776 (30.9)	386 (25.8)	318 (28.5)	<0.001	0.076
Dyslipidemia	1353 (53.9)	809 (54.0)	631 (56.5)	0.308	0.035
COPD	207 (8.3)	88 (5.9)	80 (7.2)	0.020	0.062
CAD	769 (30.6)	361 (24.1)	348 (31.2)	<0.001	0.094
Post MI	116 (4.6)	57 (3.8)	52 (4.7)	0.419	0.028
Previous PMI	106 (4.2)	67 (4.5)	84 (7.5)	<0.001	0.094
Post CABG	607 (24.2)	318 (21.2)	301 (27.0)	0.003	0.090
PAD	271 (10.8)	154 (10.3)	158 (14.2)	0.004	0.079
Atrial fibrillation	599 (23.9)	335 (22.4)	213 (19.1)	0.006	0.078
Previous stroke	253 (10.1)	155 (10.3)	123 (11.0)	0.112	0.020
STS score, %	8.0 ± 6.8	8.2 ± 6.4	7.7 ± 6.0	0.182	0.049
NYHA class Ⅲ/Ⅳ	692 (27.6)	380 (25.4)	285 (25.5)	0.221	0.033
eGFR, mL/min/1.73m^2^	51.4 ± 18.2	51.8 ± 18.2	51.3 ± 17.1	0.775	0.017
Aortic valve area, cm^2^	0.68 ± 0.20	0.64 ± 0.19	0.65 ± 0.18	<0.001	0.144
Indexed AVA, cm^2^/m^2^	0.46 ± 0.14	0.44 ± 0.13	0.46 ± 0.13	<0.001	0.109
Mean pressure gradient, mmHg	43.5 ± 15.7	49.2 ± 17.4	46.0 ± 15.6	<0.001	0.232
Bicuspid valve	81 (3.2)	87 (5.8)	10 (0.9)	<0.001	0.188
CT variables					
Annulus size of area, mm^2^	416.1 ± 78.7	405.5 ± 79.0	390.4 ± 65.8	<0.001	0.232
Annulus size of perimeter, mm	73.8 ± 6.9	72.7 ± 6.9	71.4 ± 5.9	<0.001	0.246
Procedural variables					
Transfemoral	2375 (94.7)	1442 (96.3)	1087 (97.4)	<0.001	0.094
Predilation	394 (15.7)	1194 (79.7)	1065 (95.4)	<0.001	1.616
Postdilation	418 (16.7)	176 (11.7)	100 (9.0)	<0.001	0.155
Intraprocedural antithrombotic therapy					
Any antithrombotic agent	1405 (56.0)	860 (57.4)	710 (63.6)	<0.001	0.155
Antiplatelet agents					
Aspirin	574 (22.9)	343 (22.9)	320 (28.7)	<0.001	0.133
Clopidogrel	312 (12.4)	215 (14.4)	239 (21.4)	<0.001	0.239
Prasugrel	107 (4.3)	75 (5.0)	56 (5.0)	0.447	0.036
Cilostazol	59 (2.4)	31 (2.1)	34 (3.0)	0.261	0.062
Anticoagulant agents					
Warfarin	52 (2.1)	25 (1.7)	14 (1.3)	0.212	0.064
Apixaban	117 (4.7)	72 (4.8)	55 (4.9)	0.938	0.012
Edoxaban	303 (12.1)	176 (11.7)	102 (9.1)	0.031	0.095
Rivaroxaban	45 (1.8)	32 (2.1)	21 (1.9)	0.743	0.025
Dabigatran	12 (0.5)	2 (0.1)	1 (0.1)	0.054	0.073
New-onset AF	61 (2.4)	48 (3.2)	29 (2.6)	0.335	0.047

Data are presented as mean ± SD or n (%).

Abbreviations: AF, atrial fibrillation; AVA, aortic valve area; BMI, body mass index; BSA, body surface area; CABG, coronary artery bypass grafting: CAD, coronary artery disease; COPD, chronic obstructive pulmonary disease; CT, computed tomography; eGFR, estimated glomerular filtration rate; IPTW, inverse probability of treatment weighting; MI, myocardial infarction; NYHA, New York Heart Association; PAD, peripheral artery disease; PMI, pacemaker implantation; S3UR, SAPIEN 3 Ultra Resillia; SMD, standardized mean difference; STS, Society of Thoracic Surgeons.

**Table 2:** Baseline clinical characteristics of the study population after stabilized IPTW

	After stabilized IPTW			SMD, %
	S3UR (n = 5182)	Evolut FX (n = 5418)	Navitor (n = 5935)	
Age, y	85.1 ± 5.6	85.3 ± 5.0	85.0 ± 5.4	0.038
Sex, male	1825 (35.2)	1735 (32.0)	1854 (31.2)	0.057
BSA, m^2^	1.47 ± 0.17	1.46 ± 0.18	1.46 ± 0.16	0.050
BMI, kg/m^2^	22.5 ± 3.8	22.4 ± 3.6	22.5 ± 3.5	0.019
Clinical frailty score	3.7 ± 1.3	3.7 ± 1.2	3.7 ± 1.1	0.050
Hypertension	4112 (79.4)	4374 (80.7)	4967 (83.6)	0.072
Diabetes mellitus	1445 (27.9)	1559 (28.8)	1445 (24.3)	0.067
Dyslipidemia	2865 (55.3)	2961 (54.7)	3171 (53.3)	0.026
COPD	394 (7.6)	411 (7.6)	429 (7.2)	0.010
CAD	1515 (29.2)	1636 (30.2)	1565 (26.3)	0.057
Post MI	185 (3.6)	227 (4.2)	146 (2.5)	0.064
Previous PMI	221 (4.3)	241 (4.5)	256 (4.3)	0.006
Post CABG	1120 (21.6)	1327 (24.5)	1271 (21.4)	0.049
PAD	478 (9.2)	611 (11.3)	462 (7.8)	0.080
Atrial fibrillation	1165 (22.5)	1239 (22.9)	1553 (26.1)	0.057
Previous stroke	443 (8.5)	460 (8.5)	694 (11.7)	0.071
STS score, %	8.2 ± 7.1	8.1 ± 6.4	9.2 ± 7.0	0.110
NYHA class Ⅲ/Ⅳ	1435 (27.7)	1451 (26.8)	1754 (29.5)	0.040
eGFR, mL/min/1.73m^2^	50.0 ± 19.3	51.0 ± 17.4	51.7 ± 18.0	0.060
Aortic valve area, cm^2^	0.65 ± 0.19	0.66 ± 0.20	0.64 ± 0.18	0.097
Indexed AVA, cm^2^/m^2^	0.45 ± 0.13	0.46 ± 0.13	0.44 ± 0.12	0.104
Mean pressure gradient, mmHg	48.5 ± 18.0	46.5 ± 17.1	48.3 ± 17.2	0.079
Bicuspid valve	236 (4.5)	171 (3.2)	136 (2.3)	0.084
CT variables				
Annulus size of area, mm^2^	411.4 ± 82.9	407.1 ± 81.3	395.1 ± 58.2	0.150
Annulus size of perimeter, mm	72.1 ± 7.6	73.0 ± 7.0	71.7 ± 5.3	0.129
Procedural variables				
Transfemoral	4982 (96.1)	5186 (95.7)	5860 (98.6)	0.116
Predilation	2704 (52.2)	2634 (48.6)	2638 (44.4)	0.104
Postdilation	821 (15.8)	770 (14.2)	820 (13.8)	0.038

Data are presented as mean ± SD or n (%).

Abbreviations: AF, atrial fibrillation; AVA, aortic valve area; BMI, body mass index; BSA, body surface area; CABG, coronary artery bypass grafting: CAD, coronary artery disease; COPD, chronic obstructive pulmonary disease; CT, computed tomography; eGFR, estimated glomerular filtration rate; IPTW, inverse probability of treatment weighting; MI, myocardial infarction; NYHA, New York Heart Association; PAD, peripheral artery disease; PMI, pacemaker implantation; S3UR, SAPIEN 3 Ultra Resillia; SMD, standardized mean difference; STS, Society of Thoracic Surgeons.

### In-Hospital Stroke Incidence according to Valve Type

Before IPTW adjustment, significant differences were observed in in-hospital neurologic events among valve types (Figure 2). Any stroke occurred in 1.2% of patients treated with S3UR, compared with 4.1% in the Evolut FX group and 3.5% in the Navitor group (overall *p* < 0.001, Figure 2A). Disabling stroke was infrequent with S3UR (0.3%) but occurred more often with Evolut FX (2.0%) and Navitor (1.5%; *p* < 0.001, Figure 2C). Ischemic stroke followed a similar pattern (1.2%, 3.9%, and 3.5%, respectively; *p* < 0.001, Figure 2E). Hemorrhagic strokes and TIA were rare events (Figure 2G and I).

**Figure 2:**
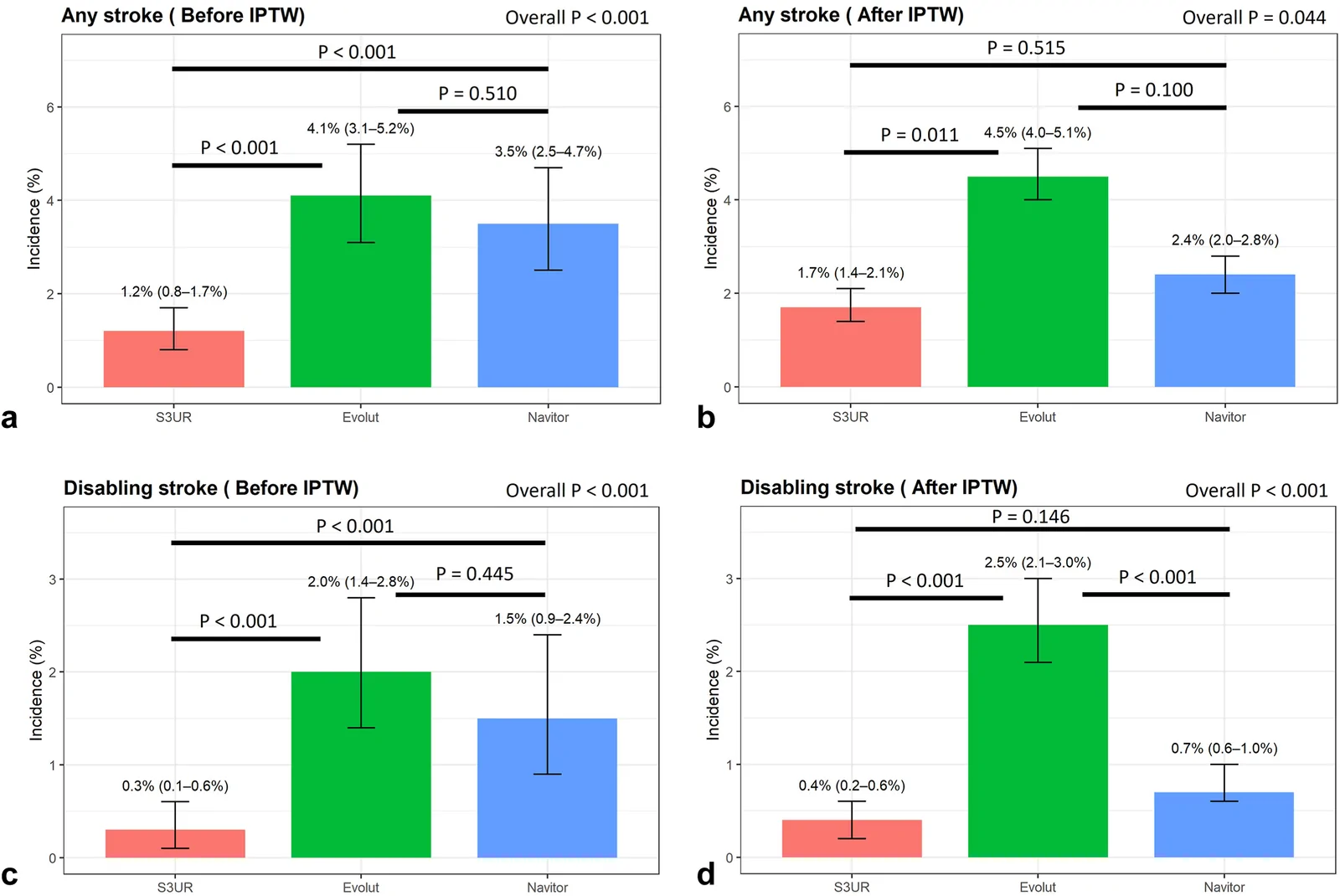

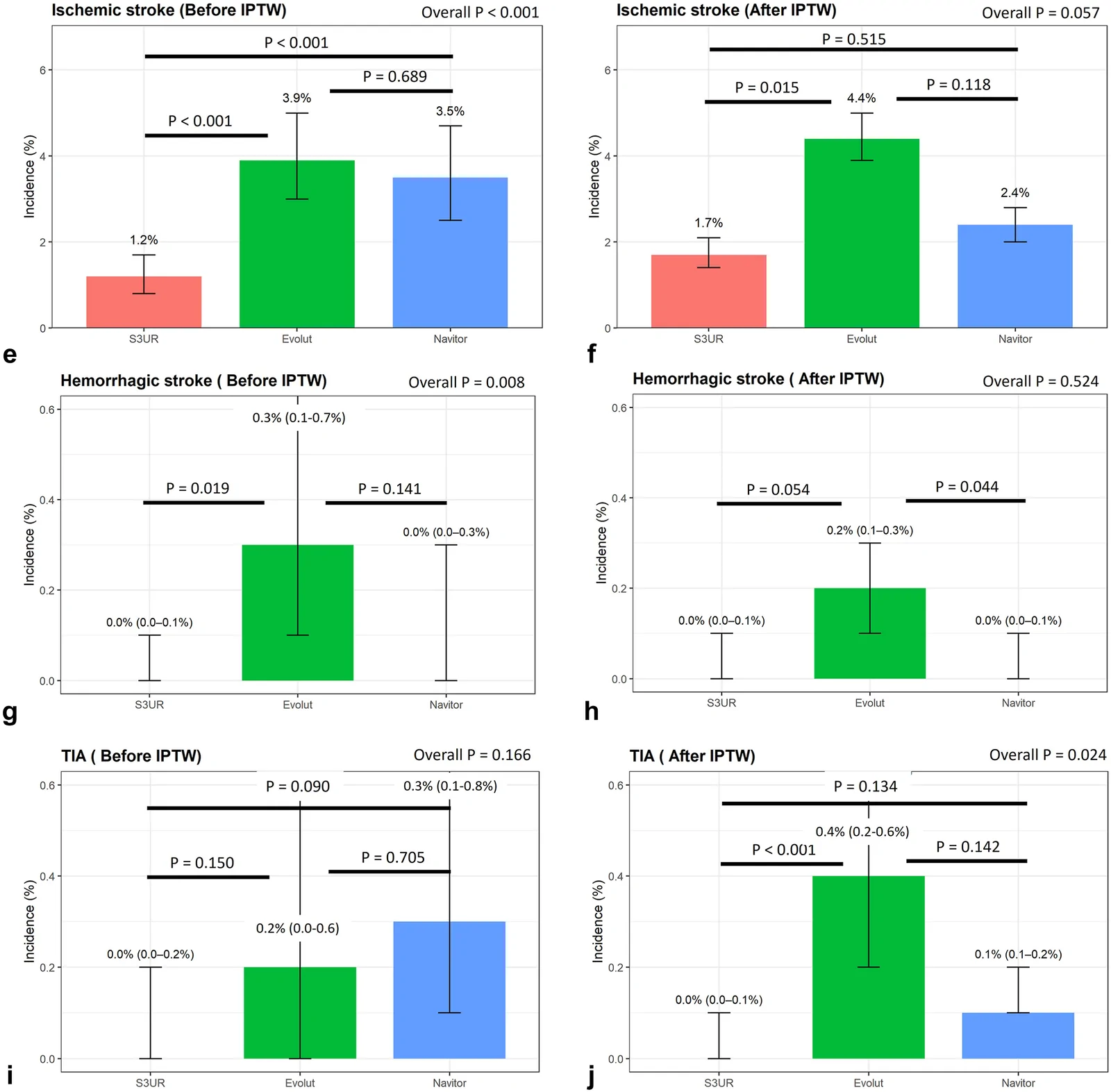
**In-hospital neurologic events after TAVR according to valve type before and after IPTW.** (a and b) Incidence of any stroke before (a) and after (b) IPTW among patients treated with S3UR, Evolut FX, and Navitor. (c and d) Incidence of disabling stroke before (c) and after (d) IPTW. (e and f) Incidence of ischemic stroke before (e) and after (f) IPTW. (g and h) Incidence of hemorrhagic stroke before (g) and after (h) IPTW. (i and j) Incidence of TIA before (i) and after (j) IPTW. Bar graphs show event rates (%) with error bars representing 95% CIs, and numeric labels indicate the incidence with corresponding 95% CI. Overall *p-*values reflect comparisons among the 3 valve groups, and pairwise *p* values compare valve types within each panel. Disabling stroke remained significantly more frequent with Evolut FX compared with S3UR and Navitor after IPTW adjustment. Abbreviations: IPTW, inverse probability of treatment weighting; S3UR, SAPIEN 3 Ultra RESILIA; TAVR, transcatheter aortic valve replacement; TIA, transient ischemic attack.

After IPTW adjustment, the weighted incidence of any stroke was attenuated but remained numerically highest with Evolut FX (1.7% with S3UR, 4.5% with Evolut FX, and 2.4% with Navitor; *p* = 0.044, Figure 2B). Importantly, disabling stroke persisted as a significant valve-specific difference after adjustment, occurring in 0.4% of S3UR patients, 2.5% of Evolut FX patients, and 0.7% of Navitor patients (*p* < 0.001, Figure 2D). Rates of ischemic stroke showed a similar directional trend after IPTW, whereas hemorrhagic stroke and TIA remained uncommon across all valve types (Figure 2F, H, and J). To account for residual covariate imbalance, doubly robust estimation was additionally performed. Results were consistent with the primary IPTW analysis: Evolut FX was associated with significantly higher odds of any in-hospital stroke (OR 2.72, 95% CI 1.46–5.07, *p* = 0.002) and disabling stroke (OR 4.27, 95% CI 1.71–10.65, *p* = 0.002) compared with S3UR (Supplemental Table 2).

### Univariable and Multivariable Predictors of In-Hospital Stroke

Univariable Firth logistic regression analyses identified valve type, nontransfemoral access, prior stroke, and predilation as factors associated with in-hospital stroke (Table 3). Specifically, compared with S3UR, both Evolut FX (OR 2.05, 95% CI 1.50–2.81; *p* = 0.00001) and Navitor (OR 1.47, 95% CI 1.01–2.10; *p* = 0.044) were associated with higher odds of stroke. Transfemoral access was independently associated with a lower risk of stroke, whereas prior stroke and predilation were associated with an increased risk. Intraprocedural antiplatelet therapy was associated with a higher incidence of in-hospital stroke (OR 1.54, 95% CI 1.09–2.18; *p* = 0.015), whereas intraprocedural anticoagulant therapy was not associated with stroke. In the multivariable Firth logistic regression model without IPTW adjustment (Table 3), valve type remained an independent predictor of in-hospital stroke. Compared with S3UR, Evolut FX (adjusted OR 2.64, 95% CI 1.67–4.16; *p* < 0.001) and Navitor (adjusted OR 2.37, 95% CI 1.35–4.18; *p* = 0.003) were associated with significantly higher odds of stroke. Additional independent predictors included lower eGFR, nontransfemoral access, and prior stroke.

**Table 3:** Univariate and multivariable firth penalized logistic regression analysis of in-hospital stroke

Variable	Univariate analysis			Multivariate analysis		
	Adjusted OR	95% CI	*p-*value	Adjusted OR	95% CI	*p-*value
Evolut FX vs. S3UR	**2.05**	**1.50–2.81**	**<0.001**	**2.64**	**1.67–4.16**	**<0.001**
Navitor vs. S3UR	**1.47**	**1.01–2.10**	**0.044**	**2.37**	**1.35–4.18**	**0.003**
Age (per year)	1.02	0.99–1.05	0.215	1.02	0.99–1.05	0.31
eGFR (per 1 mL/min increase)	0.99	0.99–1.00	0.097	0.99	**0.98–1.00**	**0.004**
TF approach	**0.49**	**0.29–0.87**	**0.016**	**0.43**	**0.24-0.81**	**0.011**
Previous stroke	**1.92**	**1.26–2.83**	**0.003**	**1.83**	**1.19–2.73**	**0.007**
DM	0.78	0.54–1.10	0.160	0.79	0.54–1.14	0.21
Predilation	1.96	1.42–2.73	<0.001	1.14	0.74–1.76	0.56
Intraprocedural antiplatelet therapy	1.54	1.09–2.18	0.015			
Intraprocedural anticoagulant therapy	0.91	0.57**–**1.40	0.68			

Bold is defined as *p* < 0.05.

Abbreviations: DM, diabetes mellitus; eGFR, estimated glomerular filtration rate; OR, odds ratio; S3UR, SAPIEN 3 Ultra RESILIA; TF, transfemoral.

### Simple Score Model Predicting In-Hospital Stroke

In the simplified score, SEV use was assigned 2 points, whereas the remaining predictors (eGFR <45 ml/min, nontransfemoral access, and prior stroke) were assigned 1 point each, yielding a total score ranging from 0 to 5 points (Supplemental Document 3). When patients were stratified into 3 groups according to the simplified score (0, 1, and ≥2 points), the incidence of in-hospital stroke increased stepwise across categories (0 points: 0.3%, 1 point: 1.8%, ≥2 points: 3.9%; *p* < 0.001), demonstrating a clear dose–response relationship (Figure 3).

**Figure 3:**
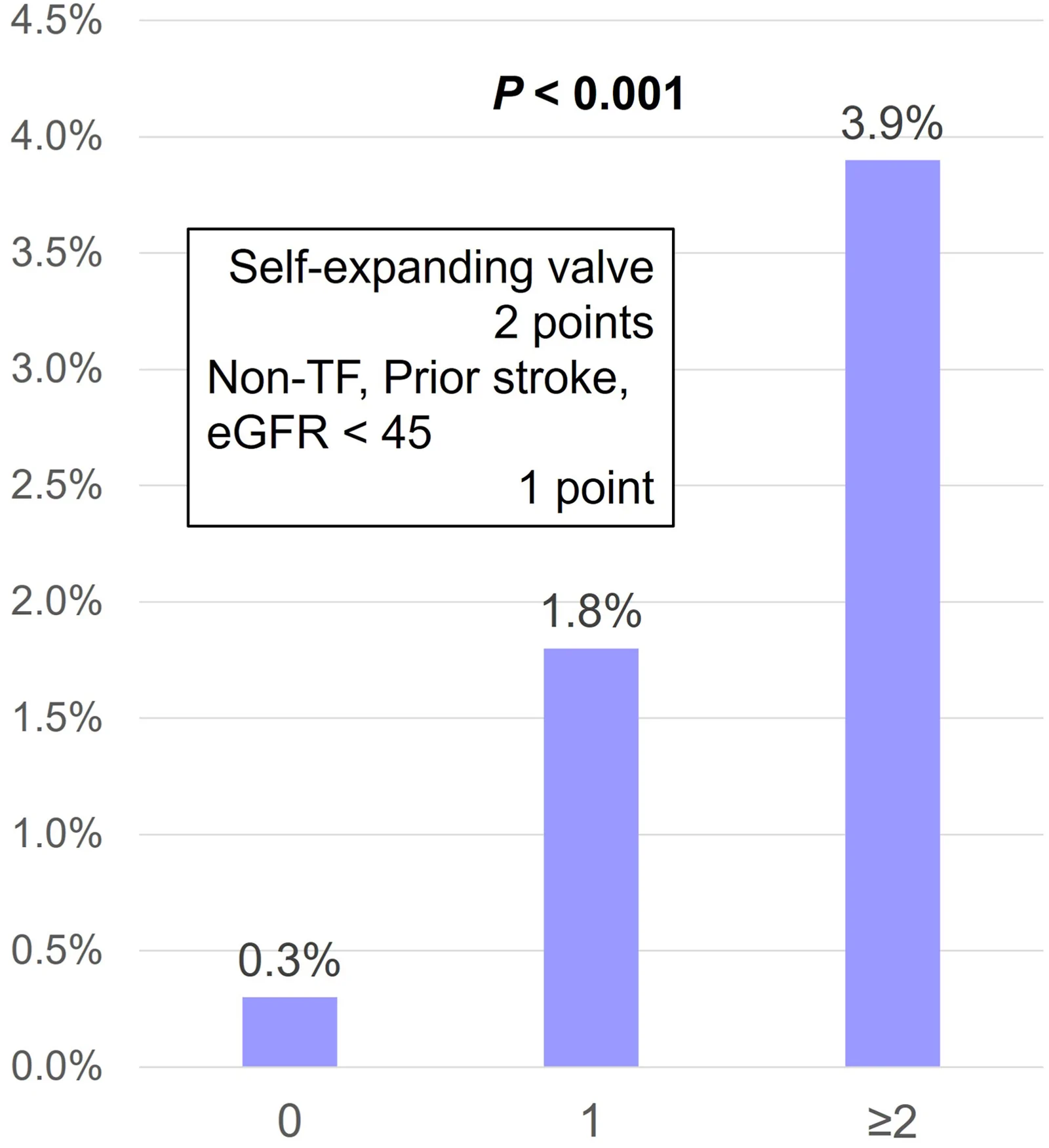
**In-hospital stroke incidence according to the simplified stroke risk score.** The incidence of in-hospital stroke increased stepwise across categories (0 points: 0.3%, 1 point: 1.8%, ≥2 points: 3.9%; *p* < 0.001). Abbreviations: eGFR, estimated glomerular filtration rate; TF, transfemoral.

### Overall Survival and Stroke Status

During follow-up, patients who experienced in-hospital stroke exhibited significantly worse survival compared with those without stroke (Figure 4). The estimated 1-year survival rate was 90.3% (95% CI: 89.4%–91.4%) in patients without stroke, compared with 73.0% (95% CI: 63.6%–83.8%) in those with in-hospital stroke (log-rank *p* < 0.001).

**Figure 4:**
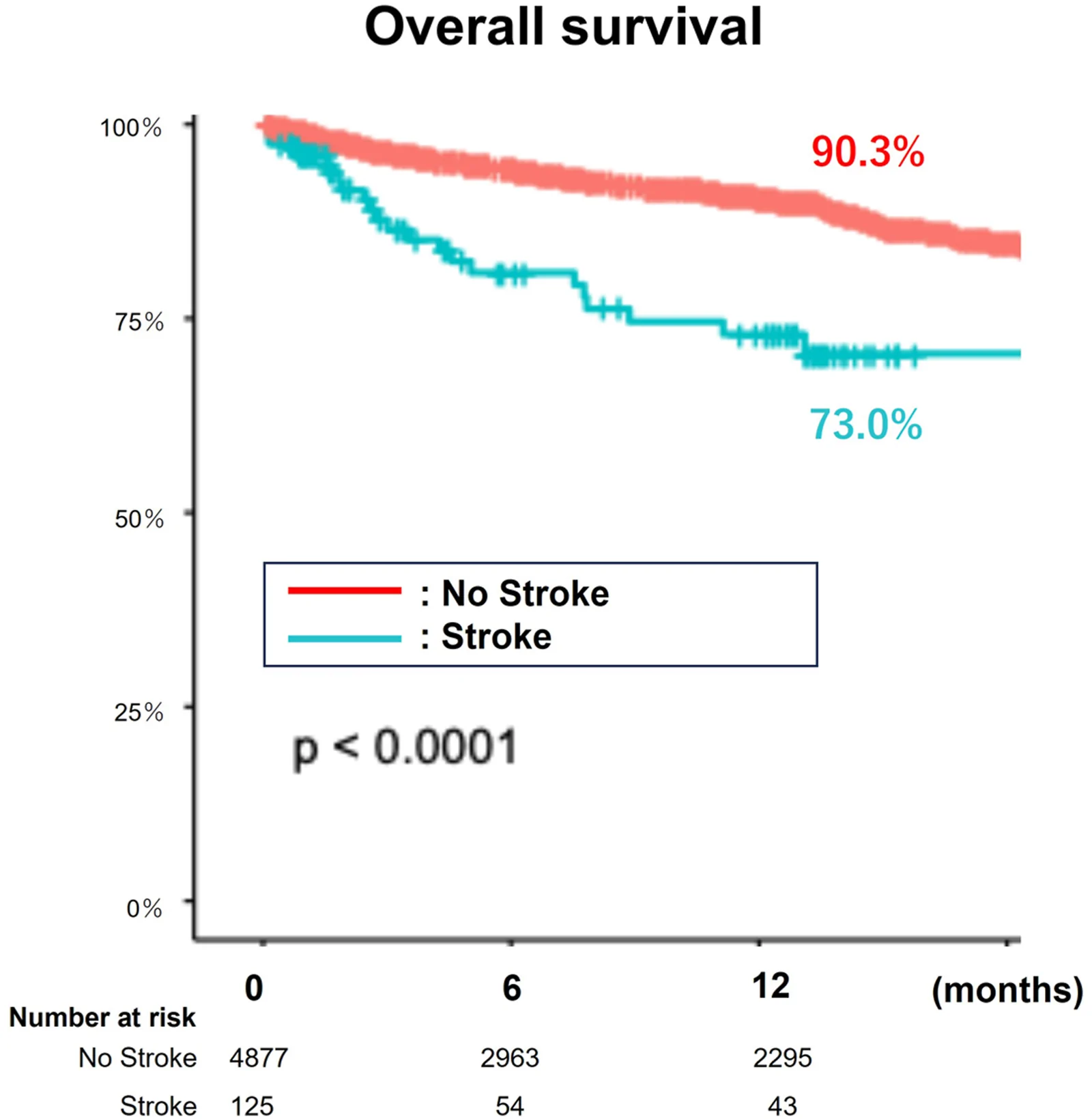
**Overall survival according to in-hospital stroke.** Kaplan–Meier curves for overall survival stratified by in-hospital stroke status. Patients who experienced in-hospital stroke had significantly worse survival compared with those without stroke. The estimated 1-year survival rate was 73.0% in patients with stroke and 90.3% in those without stroke (log-rank *p* < 0.0001). Numbers at risk are shown below the figure.

### Valve-Specific Mortality Among Patients With In-Hospital Stroke

Among patients who experienced in-hospital stroke, survival differed according to valve type (Figure 5). The estimated 1-year survival rates were 65.5% (95% CI: 46.1–93.2%) for S3UR, 81.6% (95% CI: 68.7–96.8%) for Evolut FX, and 67.4% (95% CI: 52.3–86.8%) for Navitor (log-rank *p* = 0.49).

**Figure 5:**
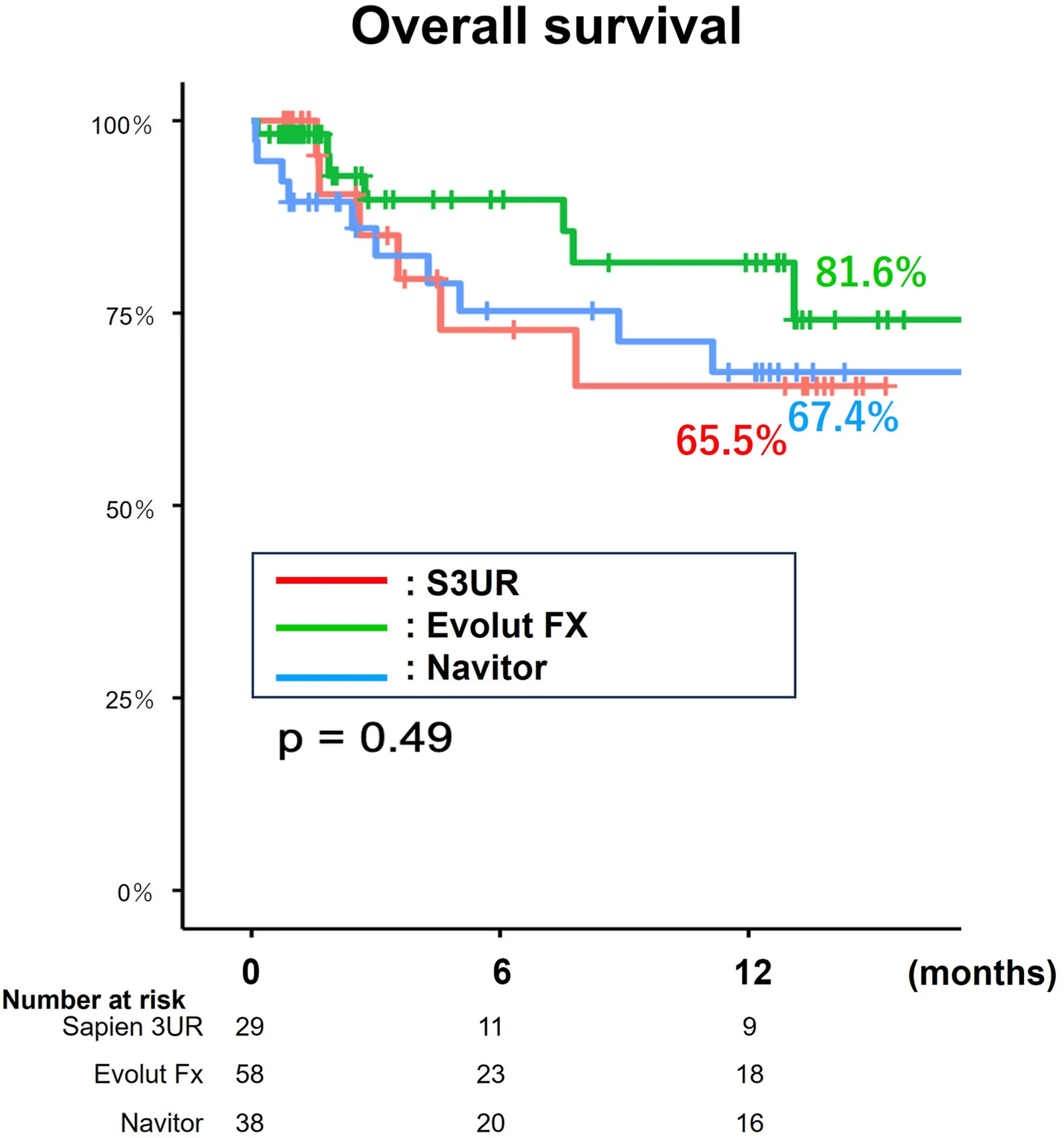
**Valve-specific survival among patients with in-hospital stroke.** Kaplan–Meier curves for overall survival among patients with in-hospital stroke, stratified by valve type. Among patients who experienced in-hospital stroke, 1-year survival rates were 65.5% for S3UR, 81.6% for Evolut FX, and 67.4% for Navitor, with no significant difference among valve types (log-rank *p* = 0.49). Numbers at risk are shown below the figure. Abbreviation: S3UR, SAPIEN 3 Ultra RESILIA.

## Discussion

In this large, contemporary TAVR cohort, in-hospital stroke remained uncommon but carried a markedly adverse prognosis. We found that in-hospital stroke following TAVR was associated with worse outcomes, and stroke risk is not uniform across transcatheter valve types. Notably, the SEVs were associated with a higher incidence of in-hospital stroke, driven largely by disabling strokes. However, this finding should be interpreted as an association rather than a causal relationship. Despite the use of IPTW to account for baseline differences, complete covariate balance was not achieved for all variables, particularly procedural characteristics such as access route and predilation. These factors may represent intrinsic components of valve-specific procedural strategies rather than confounders alone. Therefore, the observed differences in stroke incidence may reflect a combination of device characteristics and associated procedural approaches. In our multivariable Firth logistic regression model, independent predictors of in-hospital stroke included prosthesis type (SEV use), baseline renal dysfunction (lower eGFR), history of prior stroke, and use of nontransfemoral access. A simplified 3-tier risk stratification (0, 1, and ≥2 points) provided clear clinical discrimination.

Over the past decade, reported stroke rates in TAVR have declined. Early pivotal trials documented 30-day stroke incidences in the 5%–7% range, but more recent registries and trials show stabilized rates near 2%–3%. For example, the SwissTAVI registry (11,957 patients from 2011–2021) reported a 30-day stroke rate of about 3.0%.^11^ Similarly, in the recent BHF PROTECT-TAVI trial, stroke occurred in only 2.1%–2.2% of patients at 72 hours.^8^ These improvements reflect advances in valve technology, vascular access techniques, and operator experience. Stroke risk has declined over the past decade with TAVR advancements but now appears to have plateaued at around 2%–3% in current practice. In our cohort, the observed stroke incidence was in line with these figures, confirming that modern TAVR is safer than early series but has not entirely eliminated the hazard of embolic events.

Recent trials have shown that the routine use of CEP devices does not reduce overall stroke risk after TAVR. In PROTECTED TAVR, stroke occurred in 2.3% with CEP vs. 2.9% without (*p* = 0.30).^7^ The BHF PROTECT-TAVI trial found similar rates: 2.1% vs. 2.2%.^8^ Both trials were stopped for futility. Although PROTECTED TAVR noted fewer disabling strokes with CEP, this was a secondary finding. Overall, current evidence does not support routine CEP use. Registry studies suggest CEP may benefit select high-risk patients. An STS/American College of Cardiology Transcatheter Valve Therapy registry analysis showed a ∼40% reduction in disabling stroke among TAVR patients with prior stroke or TIA.^12^ This aligns with our finding that prior stroke and renal dysfunction (eGFR) were independent stroke predictors. In addition, nontransfemoral access, although less common in recent years, has consistently been associated with higher stroke risk due to greater manipulation within supra-aortic vessels. Similarly, use of SEVs, such as Evolut and Navitor, which lack active flexion mechanisms during delivery, may confer higher embolic risk during aortic arch navigation compared to balloon-expandable systems like S3UR. Although overall trials showed no benefit from routine CEP use, our results identify a subgroup of patients at higher risk of stroke—including those with prior cerebrovascular events, impaired renal function, nontransfemoral access, or SEV implantation—in whom the potential benefit of CEP remains to be established in prospective studies. This highlights the need for individualized stroke prevention strategies in TAVR. These findings may inform future risk stratification; however, their direct implications for clinical decision-making, including selective use of CEP, remain uncertain and require prospective validation.

Recent registry and post-hoc data suggest that all modern TAVR valves are associated with low stroke risk, although subtle differences exist. In a Japanese registry of small annulus cases, S3UR showed lower in-hospital stroke (0.7%) than Navitor (2.5%).^13^ A German matched analysis found no disabling strokes with Navitor versus 7.1% with Evolut.^14^ Other studies report 30-day stroke rates of 2%–3% with Evolut,^15^^,^^16^ consistent with earlier-generation SEV data. Smaller series also report low 30-day stroke rates with Navitor (0%–2.3%).^17^^,^^18^ By contrast, large U.S. registry data show no significant stroke difference between S3UR and earlier SAPIEN 3 (1.7% vs. 1.8%, *p* = 0.63).^19^ Although nonrandomized, these findings suggest S3UR have favorable stroke profile, and Navitor and Evolut may be associated with slightly higher early stroke risk.

All current TAVR platforms feature flexible delivery systems to aid arch navigation and potentially reduce embolic risk. Edwards' Commander uses a steerable flex tip, Abbott’s Navitor incorporates a hydrophilic FlexNav sheath, and Medtronic’s Evolut FX was redesigned for improved flexibility. One study found that ulcerated aortic arch plaques sharply increased 30-day stroke risk with SEVs (adjusted OR ∼9.0) but less so with balloon-expandable valves (BEVs) (OR ∼2.8), suggesting that stiff devices may dislodge debris.^20^ Although no study directly links flex mechanisms to stroke outcomes, SAPIEN’s flex design is thought to minimize trauma. Overall, the concept is biologically plausible, although not yet proven.

Our findings appear to contrast with a recent large community-practice study by Kumar et al.,^21^ which reported no significant difference in 1-year stroke rates between BEV and SEV recipients in a U.S. cohort of 6663 patients from the STS/American College of Cardiology Transcatheter Valve Therapy registry. Several important differences may explain this discrepancy. First, the endpoints differ fundamentally; our study focused on in-hospital stroke (which predominantly reflects periprocedural embolic events driven by valve deployment mechanics) whereas Kumar et al. evaluated 1-year cumulative stroke incorporating postdischarge events attributable to AF, anticoagulation management, and other nondevice factors that may attenuate any procedural signal of valve type. Second, the Kumar cohort encompassed both contemporary and potentially older-generation valve platforms under a broad BEV versus SEV classification, whereas our analysis was restricted to the latest-generation devices only. Third, the Kumar cohort exhibited significant baseline differences between valve groups, with SEV recipients being nearly 6 years older than BEV recipients (79.2 vs. 73.2 years) and carrying a higher burden of comorbidities. This raises concern for residual confounding that may have obscured a true device-specific effect despite statistical adjustment.

Finally, although no CEP device was used in our cohort, it is noteworthy that Kumar et al. themselves reported CEP use was not independently associated with 1-year stroke risk. This finding indirectly supports the notion that periprocedural stroke, rather than delayed stroke, is the endpoint most sensitive to device-specific effects and further underscores the value of evaluating in-hospital stroke as a distinct outcome in valve comparison studies.

Clinically, our findings suggested a risk-tailored approach to stroke prevention after TAVR. Despite advances in valve design, stroke remains a serious complication. Patients with prior stroke, chronic kidney disease, or nontransfemoral access face higher risk and may benefit from enhanced precautions. Although routine CEP is not supported by current evidence, whether selective use in higher-risk patients is beneficial requires prospective evaluation. In addition, subtle stroke risk differences among valve types, potentially linked to device profile and delivery system flexibility, should inform procedural planning.

### Study Limitations

Several limitations of our study merit mention. As an observational registry analysis, causal inferences are limited and residual confounding is possible despite multivariable adjustment. The absolute number of stroke events was relatively small, reflecting modern low incidence, which may have reduced the statistical power to detect some associations. In this study, in-hospital stroke was defined as any clinically apparent stroke occurring during the index hospitalization and does not correspond to the VARC-3 definition of procedure-related stroke within 72 hours after TAVR. Therefore, our findings reflect the impact of stroke events during hospitalization rather than strictly periprocedural stroke as defined by VARC-3. We also relied on site-reported neurologic outcomes without systematic neurologist adjudication. Prior work has demonstrated that routine clinical follow-up underestimates subclinical or minor strokes; studies with dedicated neurologic assessment often find 2–3 times more events than registry reports.^22^ Thus, our stroke rate might be underreported, and the true burden of silent or mild infarcts after TAVR could be higher. Detailed assessment of aortic arch and valvular plaque characteristics, including plaque composition and calcification burden on CT, was not available in this registry. Therefore, the potential impact of plaque vulnerability on periprocedural stroke risk could not be fully evaluated. Although IPTW was applied, complete balance across all covariates was not achieved, particularly for procedural variables. This residual imbalance may have influenced the observed associations and reflects the inherent limitations of observational registry data, especially in a 3-group comparison setting. In particular, Evolut FX may have been preferentially selected for anatomically complex or higher-risk patients, which could have contributed to the observed higher stroke incidence despite statistical adjustment. Intraprocedural antiplatelet therapy was administered at the operator’s discretion. Because antiplatelet agents were more likely to be used in procedures perceived to be at higher risk, the observed association with in-hospital stroke in univariable analysis likely reflects confounding by indication rather than a causal effect. Accordingly, intraprocedural antiplatelet therapy was not included in the primary multivariable model. In addition, unmeasured confounding factors, including anatomical complexity and operator preference, may have influenced valve selection and outcomes. The stroke risk score presented in this study was derived from a single-registry Japanese cohort and has not been externally validated. The generalizability of this score to non-Japanese populations may be limited given the specific characteristics of this cohort: an elderly population (mean age 85 years), small body habitus (mean body surface area, 1.45 m^2^), predominantly transfemoral access (>94%), and the near absence of bicuspid anatomy in some subgroups. These features may differ substantially from Western TAVR populations, in which patients are younger, larger, and more anatomically complex. External validation in independent cohorts, including non-Japanese registries, is warranted before clinical adoption of this score.

## Conclusions

In contemporary TAVR practice, stroke remains an infrequent but serious adverse event with identifiable risk factors. Despite advances in valve design and technique, the overall stroke rate has plateaued. Our study highlights specific predictors, such as the use of SEVs, prior stroke, renal dysfunction, and nonfemoral access, that define a higher-risk subset. These findings may inform future risk stratification strategies.

## Funding

The OCEAN-TAVI registry is supported by Edwards-Lifesciences, Medtronic, Abbott Vascular, Boston Scientific, and Daiichi-Sankyo company. The sponsors were not involved in the study conduct, data collection, statistical analysis, or manuscript writing.

## Ethics Statement

This study was conducted in accordance with the Declaration of Helsinki and institutional ethical standards. Approval was obtained from the local ethics committees of all participating centers. Because this was a retrospective analysis using anonymized clinical data, the requirement for written informed consent was waived.

## Declaration of Generative AI and AI-Assisted Technologies in the Writing Process

The authors used artificial intelligence tools to assist with language editing and manuscript preparation. All content was reviewed and approved by the authors, who take full responsibility for the integrity and accuracy of the work. The authors thank all OCEAN-TAVI investigators that participated in this study.

## Disclosure Statement

Yusuke Watanabe served as clinical proctor for and reports a relationship with Edwards Lifesciences Corporation, Medtronic, and Abbott Vascular that includes consulting or advisory. Kenji Nishioka is a clinical proctor for and reports a relationship with Edwards Lifesciences Corporation that includes consulting or advisory. Masaki Izumo is a screening proctor for and reports a relationship with Edwards Lifesciences Corporation that includes consulting or advisory. Kenichi Ishizu is a proctor of intracardiac echocardiography for and reports a relationship with Johnson & Johnson MedTech that includes consulting or advisory. Fumiaki Yashima is a clinical proctor for and reports a relationship with Medtronic that includes consulting or advisory. Hidetaka Nishina is a clinical proctor for and reports a relationship with Medtronic that includes consulting or advisory. Yohei Ohno is a clinical proctor for and reports a relationship with Medtronic and Abbott Vascular that includes consulting or advisory. Tetsuro Shimura is a clinical proctor for and reports a relationship with Medtronic and Abbott Vascular that includes consulting or advisory. Masanori Yamamoto is a clinical proctor for and reports a relationship with Edwards Lifesciences Corporation, Medtronic, and Abbott Vascular that includes consulting or advisory. Shinich Shirai is a clinical proctor for and reports a relationship with Edwards Lifesciences Corporation, Medtronic, and Abbott Vascular that includes consulting or advisory. Toru Naganuma is a clinical proctor for and reports a relationship with Edwards Lifesciences Corporation, Medtronic, and Abbott Vascular that includes consulting or advisory. Gaku Nakazawa is a clinical proctor for and reports a relationship with Edwards Lifesciences Corporation, Medtronic, and Abbott Vascular that includes consulting or advisory. Hiroshi Ueno is a clinical proctor for and reports a relationship with Edwards Lifesciences Corporation, Medtronic, and Abbott Vascular that includes consulting or advisory. Kazumasa Yamasaki is a clinical proctor for and reports a relationship with Edwards Lifesciences Corporation, Medtronic, and Abbott Vascular that includes consulting or advisory. Masahiko Asami is a clinical proctor for and reports a relationship with Edwards Lifesciences Corporation, Medtronic, and Abbott Vascular that includes consulting or advisory. Daisuke Hachinohe is a clinical proctor for and reports a relationship with Edwards Lifesciences Corporation, Medtronic, and Abbott Vascular that includes consulting or advisory. Yasushi Fuku is a clinical proctor for and reports a relationship with Edwards Lifesciences Corporation, Medtronic, and Abbott Vascular that includes consulting or advisory. Kentaro Hayashida is a clinical proctor for and reports a relationship with Edwards Lifesciences Corporation, Medtronic, and Abbott Vascular that includes consulting or advisory. The OCEAN-TAVI registry is supported by Edwards-Lifesciences, Medtronic, Abbott Vascular, Boston Scientific, and Daiichi-Sankyo company. The sponsors were not involved in the study conduct, data collection, statistical analysis, and manuscript writing.

The other authors had no conflicts to declare.

## Figures and Tables

**Supplemental Figure 1:**
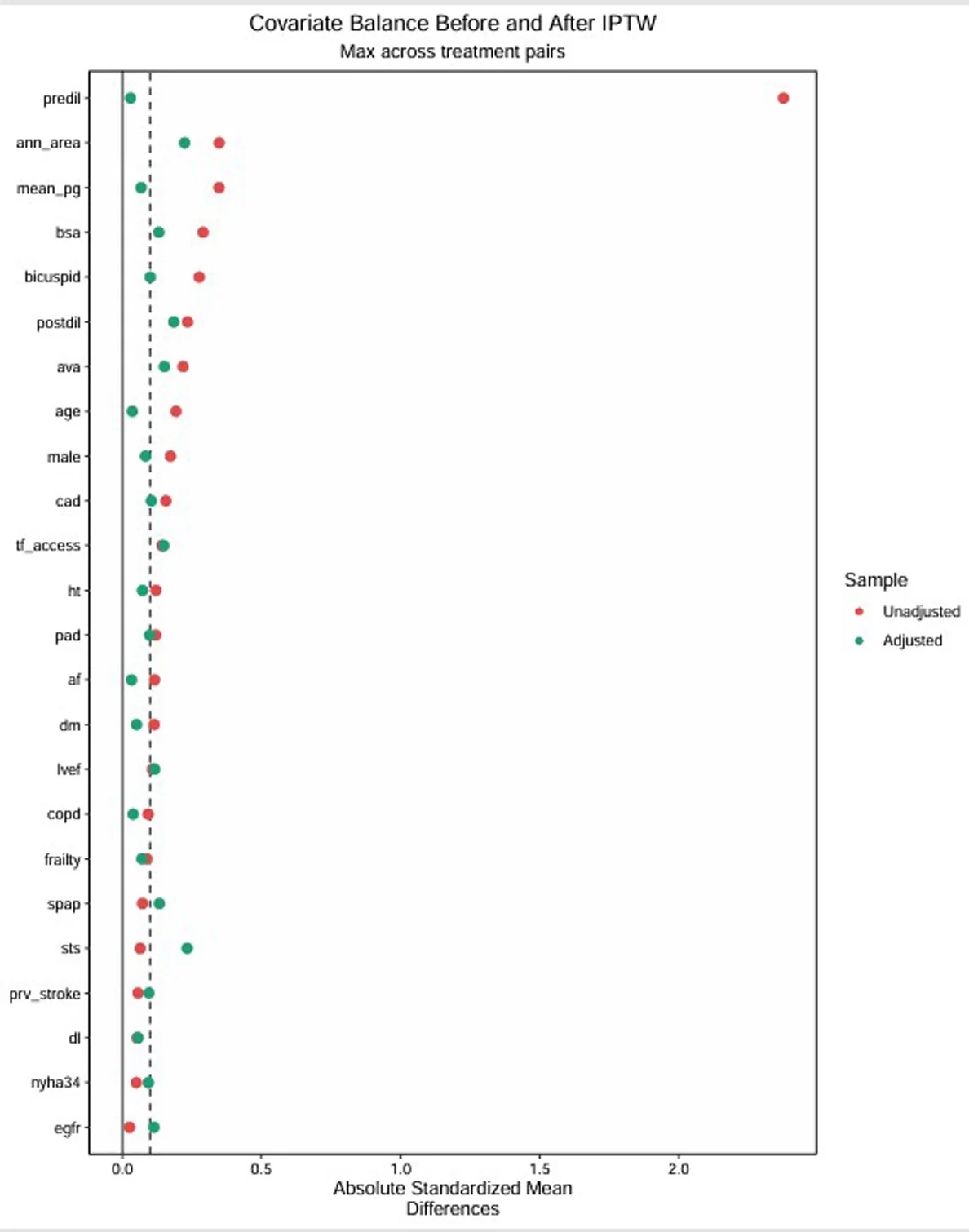
Covariate balance before and after inverse probability of treatment weighting (IPTW). Standardized mean difference (Love) plot showing absolute standardized mean differences for each covariate before (red) and after (green) IPTW. The dashed vertical line indicates the threshold of absolute SMD = 0.1. Most covariates achieved acceptable balance after IPTW adjustment. Residual imbalance in predilation was addressed by doubly robust estimation as described in the Methods. SMD, standardized mean difference

## References

[1] Gasparovic H., Tokic T. (2024). Transcatheter aortic-valve replacement in low-risk patients at five years. N Engl J Med.

[2] Mack M.J., Leon M.B., Thourani V.H. (2023). Transcatheter aortic-valve replacement in low-risk patients at five years. N Engl J Med.

[3] Yashima F., Yamamoto M., Shirai S. (2025). Initial findings concerning the latest self-expandable evolut FX valve: a report using OCEAN-TAVI registry data. Am J Cardiol.

[4] Hioki H., Yamamoto M., Shimura T. (2026). One-year clinical and hemodynamic outcomes in latest generation balloon-expandable and self-expandable transcatheter heart valves. Catheter Cardiovasc Interv.

[5] Yamanaka F., Shishido K., Moriyama N. (2025). Incidence and prognosis of prosthesis-patient mismatch after transcatheter aortic valve replacement for bicuspid aortic stenosis. JACC Cardiovasc Interv.

[6] Yamamoto M., Yashima F., Shirai S. (2024). Performance and outcomes of the SAPIEN 3 ultra RESILIA transcatheter heart valve in the OCEAN-TAVI registry. EuroIntervention.

[7] Kapadia S.R., Makkar R., Leon M. (2022). Cerebral embolic protection during transcatheter aortic-valve replacement. N Engl J Med.

[8] Kharbanda R.K., Kennedy J., Jamal Z. (2025). Routine cerebral embolic protection during transcatheter aortic-valve implantation. N Engl J Med.

[9] Lang R.M., Badano L.P., Mor-Avi V. (2015). Recommendations for cardiac chamber quantification by echocardiography in adults: an update from the American Society of Echocardiography and the European Association of Cardiovascular Imaging. J Am Soc Echocardiogr.

[10] Genereux P., Piazza N., Alu M.C. (2021). Valve academic research consortium 3: updated endpoint definitions for aortic valve clinical research. J Am Coll Cardiol.

[11] Okuno T., Alaour B., Heg D. (2023). Long-term risk of stroke after transcatheter aortic valve replacement: insights from the SwissTAVI registry. JACC Cardiovasc Interv.

[12] Butala N.M., Kapadia S.R., Secemsky E.A. (2024). Impact of cerebral embolic protection devices on disabling stroke after TAVR: updated results from the STS/ACC TVT registry. Circ Cardiovasc Interv.

[13] Mizutani K., Yamamoto M., Hioki H. (2026). SAPIEN3 ultra RESILIA versus NAVITOR valve system in patients with a small aortic annulus: insights from the OCEAN-TAVI registry. Cardiovasc Revasc Med.

[14] Campanella C., Kaiser K., Voss S. (2025). A comparison of short-term clinical outcomes between the navitor and evolut transcatheter aortic valve prostheses. J Clin Med.

[15] Carroll J.D., Mack M.J., Vemulapalli S. (2020). STS-ACC TVT registry of transcatheter aortic valve replacement. J Am Coll Cardiol.

[16] Popma J.J., Deeb G.M., Yakubov S.J. (2019). Transcatheter aortic-valve replacement with a self-expanding valve in low-risk patients. N Engl J Med.

[17] Fujita K., Matsumura K., Sugimoto K. (2025). Early clinical outcomes of transcatheter aortic valve implantation using the NAVITOR system. Cardiovasc Interv Ther.

[18] Fontana G.P., Bedogni F., Groh M. (2020). Safety profile of an intra-annular self-expanding transcatheter aortic valve and next-generation low-profile delivery system. JACC Cardiovasc Interv.

[19] Kini A.S., Tang G.H.L., Yaryura R. (2025). 1-Year real-world outcomes of TAVR with the fifth-generation balloon-expandable valve in the United States. JACC Cardiovasc Interv.

[20] Kikuchi S., Trimaille A., Carmona A. (2025). Protruding and ulcerated aortic atheromas as predictors of periprocedural ischemic stroke post-transcatheter aortic valve replacement. JACC Asia.

[21] Kumar S., Elison D., Pershad A. (2026). Stroke risk after TAVR: Balloon-expandable vs self-expanding valves in community practice. JACC Adv.

[22] Mohammed I.G., Alexandra L. (2018). Understanding neurologic complications following TAVR. Interv Cardiol.

