# Supplementary material for: Stroke After Transcatheter Aortic Valve Replacement With Contemporary Valves: Insights From the OCEAN-TAVI Registry
Source: Struct Heart. 2026 Jul 22;10(10):101099. doi: 10.1016/j.shj.2026.101099 (PMC13711442; doi:10.1016/j.shj.2026.101099)
Supplement: Supplementary file 1 [file mmc1.docx]

**Doubly robust estimation results (MICE m=20, Rubin's rules)**

| Outcome | Comparison | OR | 95% CI | p value | Interpretation |
| --- | --- | --- | --- | --- | --- |
| Any stroke | Evolut FX vs S3UR | 2.72 | 1.46–5.07 | 0.002 | Significant |
| Any stroke | Navitor vs S3UR | 1.94 | 0.85–4.43 | 0.115 | Not significant |
| Disabling stroke | Evolut FX vs S3UR | 4.27 | 1.71–10.65 | 0.002 | Significant |
| Disabling stroke | Navitor vs S3UR | 2.31 | 0.89–5.98 | 0.084 | Not significant |
