# Supplementary material for: Stroke After Transcatheter Aortic Valve Replacement With Contemporary Valves: Insights From the OCEAN-TAVI Registry
Source: Struct Heart. 2026 Jul 22;10(10):101099. doi: 10.1016/j.shj.2026.101099 (PMC13711442; doi:10.1016/j.shj.2026.101099)
Supplement: Supplementary file 2 [file mmc2.docx]

**Supplemental Methods: Inverse Probability of Treatment Weighting**

Stabilized inverse probability of treatment weighting (IPTW) was applied to account for baseline differences among patients treated with Sapien 3 Ultra RESILIA (S3UR), Evolut FX, and Navitor. Propensity scores were estimated using a multinomial logistic regression model, and stabilized weights were calculated and applied to each patient to generate a pseudo-population with balanced baseline characteristics across the three valve groups.

Covariates included all variables listed in Table 1, encompassing demographics (age, sex, body surface area [BSA], body mass index [BMI], and clinical frailty score [CFS]); comorbidities (hypertension [HT], diabetes mellitus [DM], dyslipidemia [DL], chronic obstructive pulmonary disease [COPD], coronary artery disease [CAD], prior myocardial infarction [MI], prior coronary artery bypass grafting [CABG], peripheral artery disease [PAD], atrial fibrillation [AF], and prior stroke); and markers of aortic stenosis severity (Society of Thoracic Surgeons [STS] risk score and New York Heart Association [NYHA] functional class).

Key anatomic and procedural variables were also incorporated, including aortic valve area (AVA and indexed AVA), mean transvalvular gradient, bicuspid aortic valve morphology, annular dimensions (area and perimeter assessed by computed tomography), access route (transfemoral vs. alternative), and the use of balloon aortic valvuloplasty for pre-dilation and/or post-dilation.

Missing baseline covariate data, when present, were handled using multiple imputation by chained equations prior to propensity score estimation (mice package). Covariate balance after weighting was evaluated using standardized mean differences (SMDs), with an absolute SMD <0.1 considered indicative of adequate balance between treatment groups.
